# Endoplasmic reticulum stress-regulated degradation of a translocon-associated protein is independent of integrated stress response transcription factor Gcn4p

**DOI:** 10.17912/micropub.biology.000239

**Published:** 2020-04-09

**Authors:** Kyle A Richards, Eric M Rubenstein

**Affiliations:** 1 Ball State University, Department of Biology, Muncie, IN 47306

**Figure 1:**
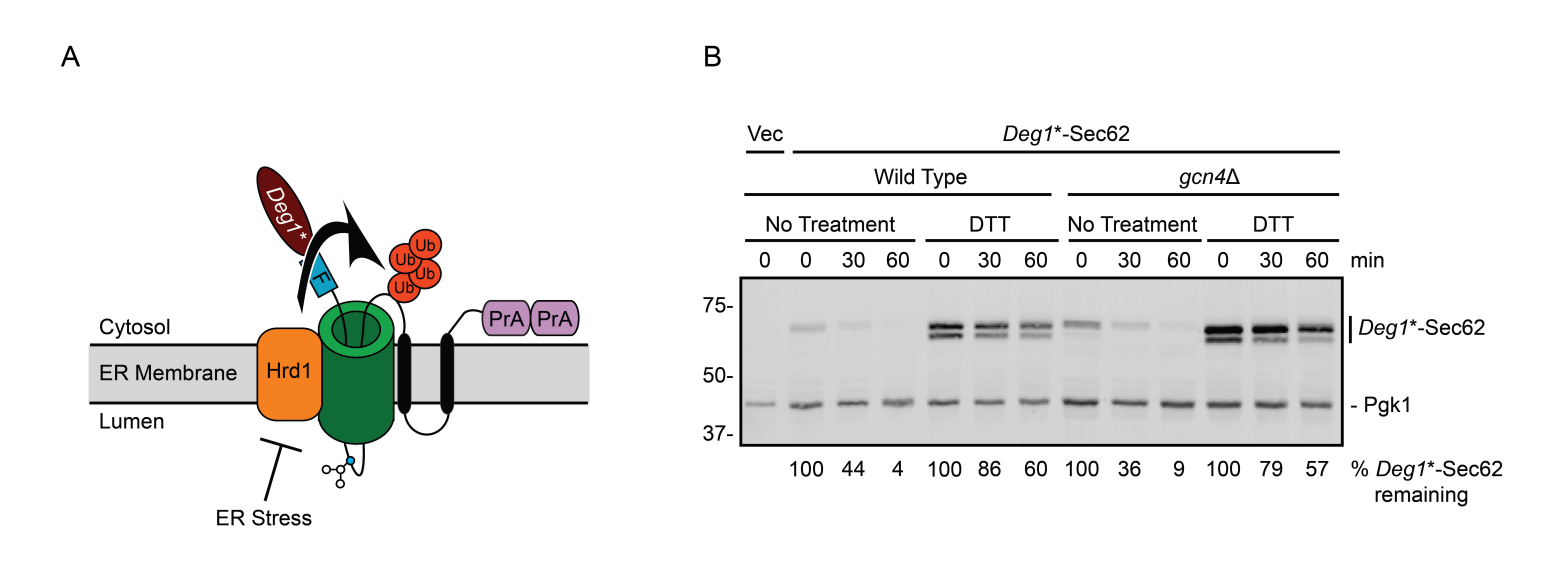
Effect of ER stress on Deg1*-Sec62 degradation in wild type and *gcn4*Δ yeast. (A) Schematic depiction of Deg1*-Sec62 following aberrant translocon engagement. Deg1*-Sec62 consists of Deg1* (a modified version of the amino-terminal 67 amino acids from the yeast transcriptional repressor MATα2p (Rubenstein *et al.* 2012)), a Flag (F) epitope, the two-transmembrane protein Sec62p, and two copies of the *S. aureus* Protein A (PrA). Following translocon engagement, Deg1*-Sec62 is modified by N-linked glycosylation and is targeted for degradation by the Hrd1p ubiquitin ligase. Degradation of Deg1*-Sec62 is impaired by ER stress (Buchanan *et al.* 2019). The primary glycosylated asparagine amino acid is portrayed as a blue circle. Ub, ubiquitin. **(B)** Cycloheximide chase of wild type and *gcn4*Δ yeast expressing Deg1*-Sec62 cultured in the absence or presence of 6 mM DTT. Cultures were treated with DTT (or water for “No Treatment” controls) for 60 min prior to cycloheximide addition; DTT was maintained at the same concentration during incubation with cycloheximide. Deg1*-Sec62 signal intensity was normalized to Pgk1, and the percentage of Deg1*-Sec62 remaining at each time point is presented below the image. This experiment was performed three times. *GCN4* deletion was confirmed by PCR. Vec, empty vector.

## Description

Most eukaryotic proteins that reside in the secretory pathway or that are released to the extracellular space cross the endoplasmic reticulum (ER) membrane by passing through the Sec61p translocon channel (Lang *et al.* 2017). Arrested translocation prevents the movement of other proteins and impairs cellular fitness (Izawa *et al.* 2012; Ast *et al.* 2016). Eukaryotes have evolved multiple quality control mechanisms to degrade proteins that aberrantly and persistently engage the translocon. Proteins that stall in the *Saccharomyces cerevisiae* translocon are targeted for proteasomal degradation by the Hrd1p (Rubenstein *et al.* 2012) or Ltn1p/Rkr1p (Crowder *et al.* 2015) ubiquitin ligases or cleaved by the Ste24p protease (Ast *et al.* 2016). These mechanisms have been intensively investigated in yeast and are likely conserved in mammalian systems (Fisher *et al.* 2011; von der Malsburg *et al.* 2015; Ast *et al.* 2016).

When misfolded or unfolded proteins accumulate in the ER, cells experience ER stress. ER stress is characteristic of a number of human diseases, and prolonged ER stress leads to cell death (Chadwick and Lajoie 2019). Further, some human cells (e.g. professional secretory cells) exist at elevated basal levels of stress (van Anken and Braakman 2005).

During ER stress in yeast, degradation of model translocon-associated substrates of Hrd1p and Ste24p is impaired (Buchanan *et al.* 2019). How ER stress impairs degradation of such proteins is not known. Impaired degradation of the Hrd1p substrate Deg1*-Sec62 (Figure 1A) during stress is not due to broadly inhibited Hrd1p function (Buchanan *et al.* 2019). Further, stress-dependent Deg1*-Sec62 stabilization does not require three established ER stress response mechanisms (unfolded protein response, ER surveillance, or stress-induced homeostatically regulated protein degradation) or one predicted ER stress response pathway (rapid ER stress-induced export), at least non-redundantly (Babour et al. 2010; Satpute-Krishnan et al. 2014; Wu et al. 2014; Szoradi et al. 2018; Buchanan et al. 2019). Additionally, elevated expression of the multifunctional ER-resident chaperone Kar2p/BiP (Rose et al. 1989) does not restore degradation during stress, consistent with impaired degradation not being due to limited chaperone availability.

The integrated stress response (ISR) is activated by a range of conditions, including ER stress (Pakos-Zebrucka *et al.* 2016; Postnikoff *et al.* 2017). During stress in yeast, the kinase Gcn2p phosphorylates the translational initiation factor eIF2α (Hinnebusch and Natarajan 2002). eIF2α phosphorylation globally attenuates translation, while specifically upregulating synthesis of the transcription factor Gcn4p, which stimulates transcription of genes required for coping with stress (Patil *et al.* 2004; Hinnebusch 2005).

We performed cycloheximide chase experiments to test the hypothesis that the ISR transcription factor Gcn4p is required for ER stress-dependent stabilization of the Hrd1p translocon-associated substrate Deg1*-Sec62 (Figure 1B). In wild type cells, Deg1*-Sec62 was rapidly degraded in non-stress conditions. As previously reported (Buchanan *et al.* 2019), Deg1*-Sec62 was profoundly stabilized during ER stress induced by dithiothreitol (DTT), which reduces disulfide bonds. ER stress also delayed N-linked glycosylation of Deg1*-Sec62, causing the protein to migrate as two species with distinct electrophoretic mobility. Deletion of *GCN4* did not stabilize Deg1*-Sec62 in the absence of stress, nor did it restore protein degradation during stress.

Our results indicate that the ISR transcriptional regulator Gcn4p is not required for degradation of Deg1*-Sec62 or its stabilization by ER stress, unless the ISR functions redundantly with other mechanisms. The molecular details by which ER stress impedes protein degradation and the cellular consequences of reduced degradation remain active areas of investigation. We speculate that impaired destruction of translocon-associated proteins is an adaptive response to ER stress. Dampened turnover of proteins that persistently engage the translocon may slow the inward flux of new proteins into an already stressed ER.

## Methods

**Yeast and Plasmid Methods**

Yeast were cultured at 30°C in standard plasmid-selective growth media (Guthrie and Fink 2004). An empty vector and a plasmid encoding Deg1*-Sec62 driven by the *MET25* promoter were introduced to wild type yeast or congenic *gcn4*Δ yeast via lithium acetate transformation (Guthrie and Fink 2004). The genotype at the *GCN4* locus in both yeast strains was confirmed by PCR.

**Cycloheximide Chase Analysis, Cell Lysis, and Western Blotting**

Cycloheximide chase analysis was performed as described previously (Buchanan *et al.* 2016). Briefly, yeast grown to mid-exponential phase at 30°C were concentrated to 2.5 OD_600_ units/ml in fresh media. Cycloheximide was added to a final concentration of 250 μg/ml. Aliquots (2.4 OD_600_ units) were harvested immediately, 30 min, and 60 min after cycloheximide addition.

Yeast were lysed as described previously (Kushnirov 2000; Buchanan *et al.* 2016). 2.4 OD_600_ units of yeast were pelleted and suspended in 200 μl of 0.1 M NaOH. Suspended yeast were incubated for 5 min at room temperature. Yeast were pelleted, resuspended in 1X Laemmli sample buffer, and heated to 95°C for 5 min.

Lysates were subject to centrifugation to pellet insoluble material. Proteins in the soluble fraction were separated by SDS-PAGE and transferred to polyvinylidene difluoride membranes via wet transfer at 20 V for 60 min at 4°C. Membranes were blocked in 5% skim milk in Tris-buffered saline (TBS; 50 mM Tris-base, 150 mM NaCl) at 4°C overnight. Membranes were probed in a solution containing 1% skim milk in TBS with 1% Tween 20 (TBS/T) and the appropriate antibodies for 1 hour at room temperature, followed by three five-min washes in TBS/T.

Deg1*-Sec62 is C-terminally tagged with two copies of the *Staphylococcus aureus* protein A epitope (Figure 1A). Because *S. aureus* Protein A binds to mammalian immunoglobulins (Hjelm *et al.* 1972), AlexaFluor-680-conjugated rabbit anti-mouse antibody (Life Technologies, Inc; 1:40,000) was used to directly detect Deg1*-Sec62. Pgk1 was detected with mouse anti-phosphoglycerate kinase 1 (Pgk1; clone 22C5D8; Life Technologies, Inc; 1:20,000) followed by AlexaFluor-680-conjugated rabbit anti-mouse secondary antibody (1:40,000). Membranes were imaged and analyzed using an Odyssey CLx Infrared Imaging System and Image Studio Software (Li-Cor).

## Reagents

The plasmids used in this study were pVJ27/pRS316 (*URA3*/CEN/ampR (Sikorski and Hieter 1989)) and pVJ411 (*LEU2*/CEN/ampR/*P_MET25_*–Deg1*-Sec62 (Buchanan *et al.* 2019)). The yeast strains used in this study were VJY476 (BY47471 *MATa his3*Δ*1 leu2*Δ*0 met15*Δ*0 ura3*Δ*0*) and VJY779(*MATa his3*Δ*1 leu2*Δ*0 met15*Δ*0 ura3*Δ*0 gcn4*Δ*::kanMX4*)(Tong *et al.* 2001).
